# Case Report: Unicentric Castleman disease mimicking metastatic lymphadenopathy in a patient with a history of triple-negative breast cancer and angiomatoid fibrous histiocytoma

**DOI:** 10.3389/fonc.2026.1985669

**Published:** 2026-10-23

**Authors:** Min He, Jing Fu, Haiyan Wang

**Affiliations:** Department of Medical Ultrasound, Shandong Medicine and Health Key Laboratory of Abdominal Medical Imaging, The First Affiliated Hospital of Shandong First Medical University & Shandong Provincial Qianfoshan Hospital, Jinan, China

**Keywords:** angiomatoid fibrous histiocytoma, differential diagnosis, histopathology, metastatic lymphadenopathy, triple-negative breast cancer, unicentric Castleman disease

## Abstract

**Background:**

Castleman disease (CD) is a rare and highly heterogeneous group of lymphoproliferative disorders. Its imaging manifestations may overlap with those of metastatic lymphadenopathy associated with malignant tumors, making the differential diagnosis of newly detected lymph node abnormalities particularly challenging in patients with a history of malignancy. Angiomatoid fibrous histiocytoma (AFH) is a rare intermediate soft tissue tumor, and the occurrence of CD in patients with a prior history of AFH has rarely been described.

**Case summary:**

We report a 38-year-old Asian woman with prior left-sided triple-negative breast cancer treated with radical surgery and adjuvant therapy. Seven years later, a left post-occipital soft tissue mass was excised and diagnosed as AFH with an *EWSR1* gene rearrangement. One year later, she noticed newly enlarged left cervical lymph nodes, raising concern for tumor recurrence or metastasis. Imaging revealed multiple lesions within the ipsilateral head and neck lymphatic drainage region. Excisional biopsy of a left level II cervical lymph node showed no evidence of malignancy on frozen section, while definitive histopathology established plasma cell-type Castleman disease. Subsequent ^18^F-fluorodeoxyglucose positron emission tomography/computed tomography (^18^F-FDG PET/CT) showed no definite abnormal lymphadenopathy in the thorax, abdomen, or pelvis, and biopsy of a left post-occipital lesion distinct from the excised AFH mass showed concordant CD-related changes. The disease was classified as unicentric Castleman disease (UCD) based on regional lesion distribution, laboratory and pathological findings, and the absence of overt systemic inflammatory manifestations or multiorgan involvement. Given the potential morbidity of complete excision, close surveillance was chosen. Six-month follow-up showed stable clinical and imaging findings.

**Conclusion:**

This case highlights the diagnostic challenges associated with the rare sequential occurrence of triple-negative breast cancer, angiomatoid fibrous histiocytoma, and Castleman disease. In patients with complex oncological histories, newly detected lymphadenopathy should not be presumed to indicate tumor recurrence or metastasis, as tumor-associated lymphoid changes and Castleman disease may present with overlapping clinical and imaging features. Accurate diagnosis and classification require integration of histopathological findings, lesion distribution, laboratory results, and clinical context. Furthermore, management should be individualized according to disease distribution, surgical feasibility, potential morbidity, and the patient's overall clinical condition.

## 1 Introduction

Castleman disease (CD) represents a rare and highly heterogeneous group of lymphoproliferative disorders (1). Based on the extent of lymph node involvement and clinical manifestations, CD is primarily classified into unicentric Castleman disease (UCD) and multicentric Castleman disease (MCD) (1, 2). CD may present with either unicentric or multicentric patterns of lymph node involvement, and affected lymph nodes may demonstrate increased ^18^F-fluorodeoxyglucose (^18^F-FDG) uptake on positron emission tomography/computed tomography (PET/CT) (1, 3). Due to the overlap between the imaging features of CD and metastatic lymph node involvement from malignant tumors (4), determining the nature of newly developed lymph node abnormalities remains challenging, particularly in patients with a previous history of malignancy (5, 6).

CD has been reported to coexist with a variety of disorders (7); however, its coexistence with solid malignancies appears to be uncommon and has been described predominantly in isolated case reports (5, 8). In patients with a history of rare neoplasms, newly detected lymph node abnormalities may pose a diagnostic challenge because benign reactive changes and metastatic disease can show overlapping imaging features (9, 10). AFH is a rare intermediate soft tissue tumor characterized by recurrent *EWSR1*-related gene rearrangements (11, 12). Only isolated reports have described CD occurring in patients with AFH or Castleman-like lymphadenopathy associated with AFH, and the potential biological relationship between these entities remains unclear (10, 13).

Herein, we report a case of a patient with a history of left-sided triple-negative breast cancer treated with radical surgery, who was subsequently diagnosed with AFH and developed new ipsilateral head and neck lymph node abnormalities during long-term follow-up. Given the patient’s history of malignancy and AFH, the imaging findings posed a diagnostic challenge in distinguishing tumor recurrence or metastasis from CD. Histopathological examination confirmed plasma cell-type Castleman disease. Further assessment of the lesion distribution, systemic manifestations, and laboratory findings supported its classification as UCD. In patients with complex oncological histories, newly developed lymph node abnormalities should therefore not be assumed to represent tumor recurrence or metastasis solely on the basis of a previous malignancy and imaging findings.

## 2 Case presentation

The patient was a 38-year-old Asian woman with a history of left-sided triple-negative breast cancer. She underwent modified radical mastectomy with axillary lymph node dissection, followed by adjuvant chemotherapy (8 cycles) and radiotherapy (25 fractions). The detailed chemotherapy regimen was unavailable because the initial treatment was performed at another institution and complete treatment records could not be retrieved. During routine postoperative surveillance over the following seven years, no evidence of local recurrence or distant metastasis was identified. The patient reported no significant family history of malignancy or other hereditary cancer-related conditions. Genetic counseling and germline testing were not performed during the current evaluation, and the potential contribution of an underlying hereditary predisposition therefore remains unknown.

Seven years after breast cancer surgery, a left post-occipital soft tissue mass was excised at another institution and diagnosed as angiomatoid fibrous histiocytoma (AFH) with *EWSR1* rearrangement. Detailed surgical information regarding the AFH lesion was unavailable. Approximately one year after AFH excision, the patient noticed enlarged left cervical lymph nodes and sought medical attention. These cervical lymph node abnormalities posed a diagnostic challenge, requiring careful differentiation from tumor recurrence or metastatic disease. The overall clinical course is summarized in **Table S1**. Imaging examinations revealed multiple enlarged lymph nodes in the left cervical region, together with newly detected nodular abnormalities in the left post-occipital region (**Figures 1A–D**). These lesions were distributed within the ipsilateral head and neck lymphatic drainage region. The nature of these lesions could not be determined solely based on imaging findings. Based on physical examination and ultrasonographic evaluation, the most prominent left level II cervical lymph node was selected for excisional biopsy. Intraoperative frozen-section analysis revealed no evidence of malignancy and did not support lymph node metastasis from breast cancer.

**Figure 1:**
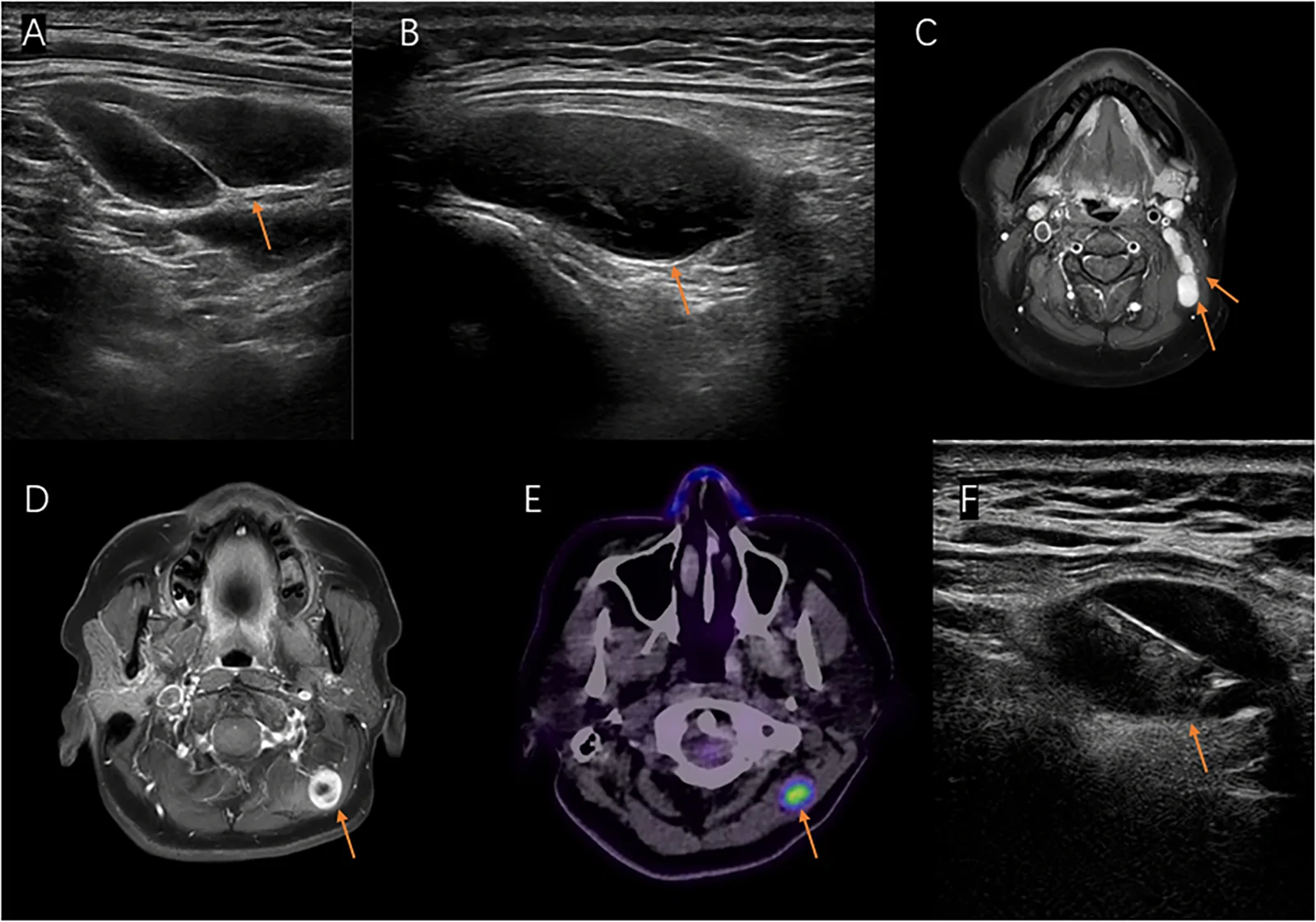
Imaging findings and biopsy procedure of Castleman disease lesions. **(A)** Ultrasonography showing the largest enlarged lymph node in the left cervical level II region (arrow). **(B)** Ultrasonography demonstrating the left post-occipital lymph node selected for further evaluation (arrow). **(C)** Contrast-enhanced T1-weighted fat-suppressed magnetic resonance imaging (MRI) showing multiple lymph nodes in the left cervical level II region (arrows). **(D)** Contrast-enhanced T1-weighted fat-suppressed MRI demonstrating the left post-occipital lymph node (arrow). **(E)** ^18^F-FDG PET/CT showing a hypermetabolic left post-occipital lymph node (arrow), with a maximum standardized uptake value (SUVmax) of 13.3. **(F)** Ultrasound-guided core needle biopsy of the left post-occipital lymph node (arrow).

Conventional histopathological examination demonstrated abnormal lymph node architecture, with atypical lymphoid follicle formation and prominent interfollicular plasma cell proliferation. Immunohistochemical staining showed that CD3 and CD20 highlighted T-cell and B-cell components, respectively, while CD21 demonstrated the follicular dendritic cell (FDC) network. Increased plasma cell components were identified by CD38 staining. Both kappa and lambda light chains showed positive expression, indicating polyclonal plasma cell proliferation. Epstein–Barr virus-encoded RNA (EBER) *in situ* hybridization was negative. Based on the morphological features and immunophenotypic profile, the lesion was diagnosed as plasma cell-type CD (**Figure 2**).

**Figure 2:**
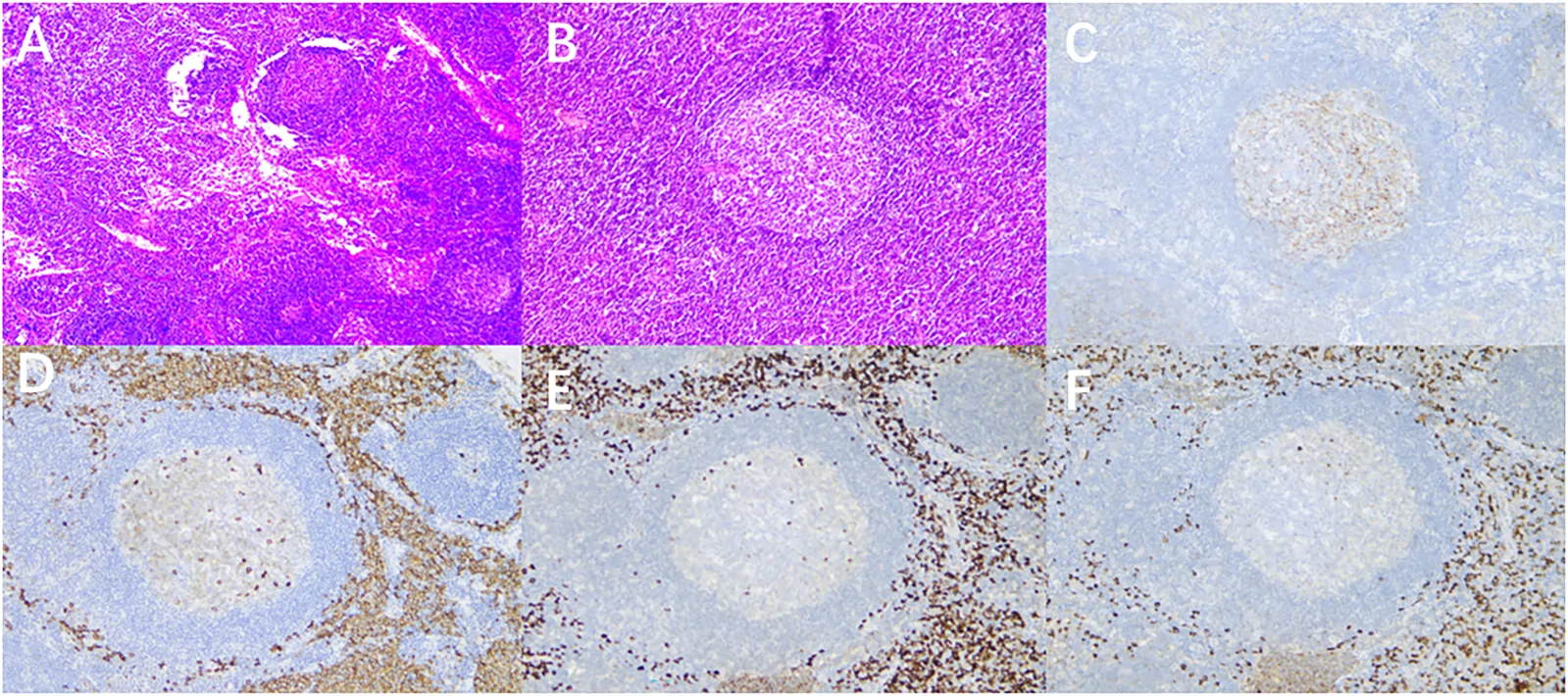
Histopathological and immunohistochemical features of the excised left cervical level II lymph node consistent with plasma cell-type Castleman disease. **(A)** Low-magnification hematoxylin and eosin (H&E) staining of the excised left cervical level II lymph node showing the overall lymph node architecture. **(B)** H&E staining (×100 magnification) showing regressed germinal center-like structures with concentric (“onion-skin”) arrangement of small lymphocytes and prominent penetrating vessels. **(C)** CD21 immunostaining demonstrating preserved follicular dendritic cell (FDC) networks within lymphoid follicles. **(D)** CD38 immunostaining showing abundant plasma cell infiltration surrounding lymphoid follicles. **(E, F)** Kappa and lambda light-chain immunostaining demonstrating expression of both light chains, supporting a polyclonal plasma cell population. CD138 staining was performed on the post-occipital biopsy specimen but was not included in this figure.

To further evaluate disease extent and assist in subtype classification, the patient underwent ^18^F-FDG PET/CT. The examination revealed multiple nodal lesions involving the left cervical and left post-occipital regions, with increased ^18^F-FDG uptake observed in some lesions (SUVmax, 2.9–13.3). No definite abnormal lymphadenopathy suggestive of CD was identified in the thoracic, abdominal, or pelvic regions, suggesting that the abnormal lymphadenopathy was predominantly confined to the left head and neck lymphatic drainage region. An ultrasound-guided core needle biopsy was subsequently performed for a newly detected metabolically active left post-occipital lymph node (SUVmax, 13.3) that was distinct from the previously excised left post-occipital AFH mass (**Figures 1E, F**). No AFH-like tumor component was identified in the sampled tissue. Histopathological examination demonstrated Castleman disease-related changes consistent with those observed in the cervical level II lymph node, and the immunohistochemical findings further supported the diagnosis of Castleman disease involvement.

Following histopathological diagnosis of Castleman disease and assessment of disease extent, further clinical and laboratory evaluations were performed for disease classification. The patient had no B symptoms, including fever, night sweats, or weight loss, and no manifestations such as skin rash, dyspnea, or peripheral neuropathy. Laboratory tests revealed no significant abnormalities in complete blood count, including anemia, thrombocytopenia, or leukocytosis. C-reactive protein (CRP) was mildly elevated (19.6 mg/L; reference range, 0–3.48 mg/L), and the serum interleukin-6 (IL-6) level was increased (29.448 pg/mL; reference range, 0–9.99 pg/mL). Serum protein electrophoresis showed a mild increase in the γ-globulin fraction without evidence of significant monoclonal immunoglobulin proliferation. Virological and immunological evaluations demonstrated undetectable Epstein–Barr virus DNA, negative human immunodeficiency virus (HIV) infection markers, normal serum IgG4 levels, and no significant abnormalities in autoimmune antibody testing.

Considering the clinical manifestations, laboratory findings, lesion distribution on imaging, and pathological characteristics, the patient showed no overt systemic inflammatory syndrome or multiorgan involvement associated with MCD, despite mild CRP elevation and an increased serum IL-6 level. Furthermore, the Castleman disease-related lesions were considered regionally distributed within the left head and neck lymphatic drainage region, with no definite abnormal lymphadenopathy suggestive of CD identified in the thoracic, abdominal, or pelvic regions. Taken together, these findings supported the classification of the disease as UCD.

### 2.1 Histopathological findings and immunohistochemistry

Excisional biopsy of the left level II cervical lymph node showed histopathological findings consistent with plasma cell-type Castleman disease. Immunohistochemistry demonstrated CD3- and CD20-positive T- and B-cell components, respectively, with CD21 highlighting the follicular dendritic cell network and CD38 showing prominent plasma cell components. Both kappa and lambda light chains were expressed without apparent light-chain restriction, supporting polyclonal plasma cell proliferation, and EBER *in situ* hybridization was negative.

Core needle biopsy of the newly detected left post-occipital lesion revealed regressed lymphoid follicles with prominent interfollicular mature plasma cell infiltration. Immunohistochemistry showed CD3- and CD20-positive lymphoid components, a CD21-positive follicular dendritic cell network, and increased CD38- and CD138-positive plasma cells. Kappa and lambda light chains were both expressed, supporting polyclonal plasma cell proliferation. In conjunction with the cervical lymph node findings, the pathological features favored plasma cell-type Castleman disease.

### 2.2 Treatment and follow-up

Following comprehensive clinical assessment, no additional therapeutic intervention was initiated, and the patient was managed with close surveillance. Although complete surgical excision represents the preferred treatment approach for resectable UCD, complete excision was not pursued because the lesions involved multiple lymph nodes within the same left head and neck lymphatic drainage region, and achieving complete excision would have required more extensive surgery. Considering the absence of systemic inflammatory manifestations, multiorgan involvement, or radiological progression, close surveillance was selected after comprehensive clinicopathological assessment. Follow-up included regular clinical assessment, laboratory monitoring, and imaging evaluation as clinically indicated. Laboratory tests included complete blood count, liver and renal function, serum albumin, and C-reactive protein, with serum interleukin-6 measured when necessary. Contrast-enhanced CT or ^18^F-FDG PET/CT was used to assess changes in lesion size, distribution, and metabolic activity. After 6 months of follow-up, the patient remained clinically stable, with no radiological progression or newly developed systemic inflammatory manifestations. Long-term surveillance was planned, with follow-up intervals adjusted according to the patient’s clinical condition.

## 3 Discussion

In this case report, we describe a rare case of plasma cell-type UCD in a patient with a history of left-sided triple-negative breast cancer and AFH. During long-term follow-up, newly detected ipsilateral head and neck lymph node abnormalities posed a diagnostic challenge, particularly in distinguishing tumor recurrence or metastatic disease from CD. Histopathological examination confirmed plasma cell-type Castleman disease, while the final classification as UCD was established through comprehensive assessment of the clinical manifestations, laboratory findings, imaging distribution, and pathological features. This case highlights the diagnostic complexity of newly detected lymph node abnormalities in patients with complex oncological histories and underscores the importance of integrating imaging, histopathology, and disease distribution for accurate diagnosis and classification.

CD exhibits heterogeneous clinical and imaging manifestations, which can complicate its distinction from other causes of lymphadenopathy (1, 4). Affected lymph nodes may show increased ^18^F-FDG uptake and imaging features that overlap with metastatic lymphadenopathy (3), limiting the specificity of imaging findings, particularly in patients with a history of malignancy (4, 6). Although CT, MRI, and ^18^F-FDG PET/CT are valuable for evaluating lesion distribution and metabolic activity, imaging findings alone cannot reliably distinguish CD from metastatic lymph adenopathy or other lymphoid disorder (3, 14). In this patient, the history of triple-negative breast cancer and AFH further complicated the interpretation of newly detected lymph node abnormalities. Excisional biopsy of the cervical lymph node provided definitive histopathological evidence of CD, while subsequent PET/CT delineated the distribution of the lesions and biopsy of the post-occipital lesion demonstrated concordant CD-related pathological changes. These findings underscore the complementary roles of imaging and histopathological assessment in the diagnosis and classification of CD.

CD has been reported to coexist with a range of clinical disorders, and its co-occurrence with hematological malignancies, particularly lymphoma, has been more extensively documented (15). In contrast, synchronous or metachronous occurrence of CD with solid malignancies appears to be uncommon, with the available evidence consisting largely of isolated case reports (8, 16, 17). These reports document clinical coexistence but do not establish a causal relationship, and the biological basis of any association between CD and solid malignancies remains uncertain (8, 16, 17).

The pathogenesis of CD appears to differ between UCD and MCD. In MCD, particularly idiopathic multicentric Castleman disease (iMCD), systemic cytokine dysregulation is a prominent feature, with IL-6 contributing substantially to inflammatory manifestations in many patients; in contrast, UCD is generally localized, and its underlying pathogenesis remains incompletely understood (1, 7). Increasing evidence suggests that abnormalities of the local lymph node stromal microenvironment, particularly involving follicular dendritic cells (FDCs) and other stromal cells, may contribute to the development of UCD (1, 18). Recurrent activating *PDGFRB* mutations have also been identified in CD45-negative stromal cells in a subset of UCD cases, providing molecular support for a stromal-cell–associated mechanism in at least some patients (19). Although IL-6-related inflammatory abnormalities may occur in a minority of patients with UCD, particularly those with plasmacytic histopathology, systemic inflammatory activity is generally less prominent than in MCD (1). In the present case, despite mild elevations in CRP and serum IL-6, the absence of an overt systemic inflammatory syndrome or multiorgan involvement, together with the absence of widespread multicentric lymphadenopathy and the regional distribution of CD-related lesions within the left head and neck lymphatic drainage region, supported the classification of UCD rather than MCD (1, 2).

AFH is a rare intermediate soft tissue tumor characterized by recurrent *EWSR1*-related gene rearrangements, most commonly involving *EWSR1-CREB1*, with *EWSR1-ATF1* and *FUS-ATF1* occurring less frequently (11, 12). In addition to its characteristic molecular alterations, AFH may exhibit prominent lymphoplasmacytic inflammation, and a subset of cases, particularly those harboring *EWSR1-CREB1* fusion, has been associated with excessive IL-6 production and paraneoplastic inflammatory manifestations (20, 21). Rare reports have described Castleman-like lymph node changes in association with AFH; notably, an early case demonstrated plasma cell-type Castleman-like lymphadenopathy that regressed substantially after resection of the primary AFH, supporting a reactive process, while a subsequent report documented the coexistence of AFH and Castleman disease (10, 13). These observations suggest a possible cytokine-mediated or immune-related overlap between AFH and CD, particularly involving IL-6-related inflammatory signaling; however, such a relationship remains speculative. Current evidence is limited to isolated reports, and no direct molecular link between AFH-associated EWSR1 rearrangements and the development of CD has been established (10, 13, 20). Therefore, the occurrence of UCD following AFH in the present patient should be regarded as an uncommon clinical observation rather than evidence of a causal relationship. In the present case, although the regional distribution of the lesions and concordant pathological findings supported coexisting UCD, the contribution of previous AFH-associated immune changes cannot be completely excluded because longitudinal pathological information regarding the original AFH lesion was unavailable.

The management strategy was also individualized according to the extent of disease and clinical status. Complete surgical excision is considered the preferred treatment approach for resectable UCD because it may provide definitive disease control and long-term remission (1). However, in the present case, the lesions involved multiple lymph nodes within the same left head and neck lymphatic drainage region rather than a single isolated resectable mass. Complete removal of all involved lesions would have required more extensive surgery, with potential additional surgical morbidity. Given the absence of systemic inflammatory manifestations, organ involvement, or radiological progression, close surveillance was considered an appropriate approach (1). During 6 months of follow-up, the patient remained clinically stable without radiological progression or newly developed systemic inflammatory manifestations.

Several limitations should be acknowledged. First, as a single case report, this study cannot establish a causal or biological relationship among angiomatoid fibrous histiocytoma, triple-negative breast cancer, and Castleman disease. Second, the original AFH lesion was treated at another institution, and the complete surgical records, pathological slides, and molecular data were unavailable for review. Therefore, the completeness of AFH excision, margin status, and local tumor extent could not be fully assessed. In addition, the newly detected post-occipital lesion was evaluated by core needle biopsy. Although no morphologic evidence of AFH was identified in the sampled tissue, the limited nature of the biopsy means that a very small focus of AFH involvement in unsampled tissue cannot be excluded with absolute certainty. Third, detailed pathological and molecular information regarding the previously treated breast cancer was unavailable, and germline genetic testing was not performed; therefore, whether an underlying hereditary predisposition contributed to the sequential occurrence of these rare diseases remains unknown. Fourth, the ancillary pathological and virological work-up was incomplete. A formal quantitative kappa/lambda ratio was not available, tissue IgG4/IgG immunostaining was not performed, and immunohistochemistry for human herpesvirus 8 (HHV-8) latency-associated nuclear antigen 1 (LANA-1) and peripheral blood HHV-8 DNA testing could not be completed. Although both kappa and lambda light chains were expressed without apparent restriction and the serum IgG4 level was normal, the absence of these additional studies limited a more comprehensive evaluation of alternative causes of plasmacytic or Castleman-like lymphadenopathy. Finally, although the patient remained clinically stable during the 6-month follow-up period, longer-term follow-up and additional well-documented cases are needed to further characterize the clinical course of CD in this setting and to clarify any potential biological relationship among these conditions.

## 4 Conclusion

This case highlights a rare and diagnostically challenging clinical scenario involving the sequential occurrence of triple-negative breast cancer, angiomatoid fibrous histiocytoma, and Castleman disease. In patients with complex oncological histories, newly detected lymphadenopathy may represent a diagnostic dilemma because malignant recurrence, metastatic disease, tumor-associated lymphoid changes, and Castleman disease can present with overlapping clinical and imaging features. This case provides additional clinical insight into the evaluation of Castleman disease-like lymphoid lesions arising in the setting of previous neoplasms. Rather than attributing new lymphadenopathy solely to prior malignancies or tumor-related immune phenomena, careful integration of pathological findings, lesion distribution, laboratory evaluation, and clinical context is essential for accurate diagnosis and classification. Furthermore, this case emphasizes the importance of individualized management in rare lymphoproliferative disorders. Treatment decisions should consider not only established disease guidelines but also lesion distribution, surgical feasibility, potential morbidity, and the patient’s overall clinical status.

## Data Availability

The datasets presented in this article are not readily available because no standalone dataset was generated for this case report. The underlying clinical data are not publicly available because they contain potentially identifiable patient information and are subject to institutional and ethical restrictions. Requests to access the datasets should be directed to Haiyan Wang, wanghaiyan96@126.com.
