# Supplementary material for: Case Report: Unicentric Castleman disease mimicking metastatic lymphadenopathy in a patient with a history of triple-negative breast cancer and angiomatoid fibrous histiocytoma
Source: Front Oncol. 2026 Oct 23;16:1985669. doi: 10.3389/fonc.2026.1985669 (PMC13739528; doi:10.3389/fonc.2026.1985669)
Supplement: Supplementary file 1 [file Table1.docx]

**Supporting Information (SI)**

Case Report: Unicentric Castleman Disease Mimicking Metastatic Lymphadenopathy in a Patient With a History of Triple-Negative Breast Cancer and Angiomatoid Fibrous Histiocytoma

Min He, Jing Fu and Haiyan Wang^*^

Department of Medical Ultrasound, Shandong Medicine and Health Key Laboratory of Abdominal Medical Imaging, The First Affiliated Hospital of Shandong First Medical University & Shandong Provincial Qianfoshan Hospital, Jinan, China

*To whom correspondence should be addressed: [wanghaiyan96@126.com](mailto:wanghaiyan96@126.com)

**Table S1. Timeline of Clinical Events**

| **Time Point** | **Clinical Event** | **Diagnostic Evaluation and Management** | **Main Findings and Clinical Significance** |
| --- | --- | --- | --- |
| Initial stage | Diagnosis of left-sided triple-negative breast cancer | Radical surgery followed by adjuvant therapy | The patient subsequently entered long-term follow-up |
| Seven years after breast cancer treatment | A left post-occipital soft tissue mass developed | Excision of the lesion followed by histopathological and molecular evaluation | Histopathological examination established the diagnosis of angiomatoid fibrous histiocytoma (AFH), and molecular testing revealed an EWSR1 gene rearrangement |
| One year after AFH diagnosis | Newly detected left cervical lymph node abnormalities during follow-up | Imaging evaluation, including ultrasonography and MRI, was performed | Multiple enlarged lymph nodes were identified in the left cervical region, together with newly detected nodular abnormalities in the left post-occipital region. Imaging findings alone could not determine the nature of the lesions, which required differentiation from tumor recurrence or metastasis |
| Same period | Further evaluation of the left cervical lymph node abnormalities | Excisional biopsy of the most prominent left level II cervical lymph node with intraoperative frozen-section analysis | Frozen-section analysis showed no evidence of malignancy and did not support lymph node metastasis from breast cancer. Definitive histopathological examination established the diagnosis of plasma cell-type Castleman disease |
| **Time Point** | **Clinical Event** | **Diagnostic Evaluation and Management** | **Main Findings and Clinical Significance** |
| Subsequently | Assessment of disease extent and subtype | ¹⁸F-FDG PET/CT was performed | Multiple nodal lesions were identified in the left cervical and left post-occipital regions, with increased 18F-FDG uptake in some lesions. No definite abnormal lymphadenopathy suggestive of Castleman disease was identified in the thoracic, abdominal, or pelvic regions |
| Same period | Further evaluation of the newly detected left post-occipital lesion | Ultrasound-guided core needle biopsy of the metabolically active left post-occipital lymph node | The lesion was distinct from the previously excised AFH lesion. Histopathological findings showed Castleman disease-related changes concordant with those of the left level II cervical lymph node, further supporting Castleman disease involvement |
| After completion of pathological and disease-extent assessment | Disease classification | Comprehensive assessment of clinical manifestations, laboratory findings, imaging distribution, and pathological features | Despite mild CRP elevation and increased serum IL-6, the patient had no overt systemic inflammatory syndrome or multiorgan involvement. The Castleman disease-related lesions showed a regional distribution within the left head and neck lymphatic drainage region, supporting classification as UCD |
| After diagnosis and classification | Management decision | No additional therapeutic intervention was initiated, and the patient was managed with close surveillance | This approach was based on the absence of overt systemic inflammatory manifestations or multiorgan involvement and the regional distribution of Castleman disease-related lesions within the left head and neck lymphatic drainage region |
| **Time Point** | **Clinical Event** | **Diagnostic Evaluation and Management** | **Main Findings and Clinical Significance** |
| Follow-up | Ongoing clinical surveillance | Regular clinical assessment, laboratory monitoring, and imaging evaluation as clinically indicated | To date, the patient remains clinically stable, with no radiological progression or newly developed systemic inflammatory manifestations |
